# George Edwin Hunt, MD., D.D.S.

**Published:** 1914-09-15

**Authors:** 


### OBITUARY.

George Edwin Hunt, md., d.d.s.—We print below an obituary notice of Dr. G. E. Hunt, of Indianapolis. We were both greatly surprised and shocked to learn of the death of Dr. Hunt. It seems only a few days since he greeted us at Rochester with the same genial and whole-souled remarks that characterized and made him the lasting friend of any one who had the pleasure of his friendship. He has been a large factor in professional affairs during all the time of his association with the profession. He had a keen mind and a charitable disposition that made him a man of preferment and influence. As a teacher, editor, and professional worker he did a tremendous lot of good work in a short life. He was devoted to his profession and the friends he made in it, and there is many a sad heart throughout the land because he has passed on to the better world. He was our friend for many years and we fail for words to express our personal loss. His memory will always be precious to us. We would join in cordial sympathy with his many other friends in extending to his wife and sisters our appreciation of the worth of his life to our profession and our great loss of a dear personal friend. We print below a more extended sketch of his life taken from *Oral Hygiene* which journal absorbed much of his time and strength and in which he was vitally interested.

Dr. George Edwin Hunt was a native of Indianapolis. Born April 29th, 1864.

His early education was in the public schools of his native city, where he completed the common schools and the first two years of high school.

Entering what is now the DePauw University at Greencastle in 1882, he completed a two year course in civil engineering after which he attended the University of Michigan at Ann Arbor for one year.

For the following four years he was engaged in railroad engineering work in Florida, after which he entered the Indiana Dental College, graduating from there in 1890 with the degree of Doctor of Dental Surgery, subsequently attending the Indiana Medical College where he received the degree of Doctor of Medicine.

Following this, Dr. Hunt engaged actively in the practice of dentistry in Indianapolis, being elected in March 1891 to the Board of Trustees of the Indiana Dental College, and was subsequently elected to the position of Secretary of that institution, and in 1896, closed his dental office and devoted his entire time to the executive work as Dean of the institution.

Dr. Hunt had been a member of the National Dental Association since 1891, and was Vice-President of the organization in 1906 and 1907. In 1904 he served as Vice-President of the Fourth International Dental Congress which met in St. Louis. He has been a member of the Indiana State Dental Association since 1890, having served seven years as Secretary, and President one year.

He became a member of the National Association of Dental Faculties in 1895 and was Secretary of that organization until 1913, at which time he was named Vice-President, and in 1914 President of the organization.

Dr. Hunt in addition to the above positions of honor, was a prominent member of the Delta Sigma Delta Fraternity, having held several important offices in same, and was also a member of the Delta Tau Delta Fraternity.

He had for the past fourteen years edited *Desmos*, the magazine of his dental fraternity, and since January, 1911, has been editor in chief of *Oral Hygiene*, to which publication, save for his untimely end, he had expected to devote his entire time henceforth.

Dr. Hunt's work as an author on subjects of interest to the dental profession, is of incalculable value and is well known to the profession.

Locally he was a member of several civic organizations, his political efforts being always in the cause of cleaner government.

He became affiliated with the Masonic Fraternity in 1885, attaining the Thirty-Second Degree of the Ancient and Accepted Scottish Rite, in addition to which he was a member of Murat Temple Ancient Arabic Order of the Nobles of the Mystic Shrine.

His death occurred at his residence in Indianapolis, Saturday, July 11th, 1914, cause attributed by physician in attendance being acute gastritis.

He is survived by his widow, Maria Foster Hunt, his step-daughter, Francis Buchannan, a single and a married sister.

In the May issue of *Demos*, a Fraternity Publication of which he had been Editor for fourteen years, George Edwin Hunt, who has edited this publication since the first issue appeared, printed an editorial entitled "Farewell to All" and in his closing paragraph, in his inimitable way said:

"My lares and penates beckon. I go to my dog and my cat; to The Woman—Who—Autos—with—me; to my life-like imitation of a truckgarden; to the dod-buttered obscurity of a life of ease after basking in the refulgent spot-light of epoch shaping editorial brilliance. But I go in peace with all the world. *Vale.*"

The task of editing two publications had become too heavy for him. He was obliged to choose between the two. And unhesitatingly decided in favor of continuing his work in this magazine.

Meanwhile for the past six months his health had failed to an extent that prompted him to say in his leading editorial for this issue of *Oral Hygiene* that now, in addition to preaching vacations to others he proposed to take his own prescriptions and that he would be "off on that vacation by the time this number is in the mails."

Unfortunately for the Profession of which he was an honored member, unhappily for those to whom he had endeared himself by his personality, by his brilliant and active mind; by his charm, his utterances were prophetic. He has gone on his vacation—but he will never return.

Returning July 9th from Rochester, where he was called by his professional duties in connection with the meeting of the National Dental Association, he complained on the following evening of severe pains in the regions of his heart, and on Saturday morning, July 11th, was found dead in his bed at his residence in Indianapolis.

So passes a man, who through the medium of this publication as well as the almost unlimited amount of correspondence incident to editing a magazine of this character, in addition to his utterances from the public rostrum, has probably done more for the benefit of humanity in general and the dental profession in particular than any man of his time.

That his work will live after him is a forgone conclusion. That he has paved the way for carrying on the work to which he was dedicated the last and four best years of his life is certain. He has but reached the end of his division. The grade will be easier and the road bed smoother for his successor.

It is a pitiful fact that he could not have lived to realize his frequently expressed ambition of seeing a free clinic established in every public school in the land at the expense of the state, and with adequate facilities for caring for the mouth of each child unable to pay for dental service but that day is coming. It may not be this year or even this generation, as radical reforms require time for their fulfillment in direct ratio with the importance with the propaganda, but when that day does come, no matter what means may be devised to that end, or by whom; no matter who may succeed him as editor of this publication, the name of the man who blazed the trail should and will be written in capital letters that spell—GEORGE EDWIN HUNT.

